# Histopathological features of subcutaneous and cutaneous mast cell tumors in dogs

**DOI:** 10.1186/s13028-024-00775-5

**Published:** 2024-10-01

**Authors:** Stella Minnoye, Shana De Vos, Samuel Beck, Luc Duchateau, Mike Hubers, Sieglinde David, Ruth Fortrie, Hilde de Rooster

**Affiliations:** 1 Anicura Dierenkliniek Randstad, Frans Beirenslaan 155, Borsbeek, 2150 Belgium; 2 Department of Morphology, Imaging, Orthopedics, Rehabilitation and Nutrition, Ghent University, Salisburylaan 133, Merelbeke, 9820 Belgium; 3 Cancer Research Institute Ghent, Medical Research Building, University Hospital Ghent, Salisburylaan 133, Merelbeke, 9820 Belgium; 4 Independent Anatomic Pathology Ltd, c/o Moore Scarrott Veterinary, Calyx House, South Road, Taunton, Somerset, TA1 3DU UK; 5 Biometrics Research Center, Ghent University, Salisburylaan 133, Merelbeke, 9820 Belgium; 6 Department of Soft Tissue Surgery, Anicura Medisch Centrum voor Dieren, MCD, Isolatorweg 45, Amsterdam, 1014 The Netherlands; 7 Anicura Dierenkliniek Hond en Kat, Emiel Clauslaan 134, Deinze, 9800 Belgium; 8 Small Animal Department, Ghent University, Salisburylaan 133, Merelbeke, 9820 Belgium

**Keywords:** Canine, Grading system, Kiupel, Neoplasm, Patnaik

## Abstract

**Background:**

Mast cell tumors (MCTs) are the most common malignant skin neoplasms in dogs. In the past, the distinction between cutaneous MCTs (cMCTs), originating from the dermis, and subcutaneous MCTs (scMCTs), originating from the subcutaneous tissue, was not made. Histopathological differentiation, including grading, is important for prognosis. However, the Patnaik and Kiupel grading systems were proposed for cMCTs only. The objective of our study was to describe and compare the signalment of dogs with scMCTs and cMCTs and histopathological features, anticipating similarities in both groups. Data of dogs histologically diagnosed with scMCTs or cMCTs between September 2020 and July 2023 were analyzed retrospectively. Signalment, tumor location, histopathological features, completeness of removal and lymph node status were recorded.

**Results:**

Data on 305 scMCTs and 1291 cMCTs were collected. Breed distribution was different between scMCTs and cMCTs (*P* < 0.0001). Mitotic count (MC) was not different between scMCTs (1.63) and cMCTs (1.58) (*P* = 0.8490). Compared to cMCTs, scMCTs more often had anisokaryosis, bizarre nuclei and multinucleation. Kiupel high grade was more often assigned to scMCTs (51/292, 17.5%) than cMCTs (154/1291, 11.9%) (*P* = 0.009). The odds of MCTs being assigned a high grade in scMCT was 1.578 higher than in cMCTs (95% confidence interval [1.116–2.232]).

**Conclusions:**

Histopathological differences between scMCTs and cMCTs were observed. A Kiupel high grade was more often assigned to scMCTs than cMCTs. Whether these histopathological findings correlate with clinical outcome has to be established in additional studies.

## Background

Mast cell tumors (MCTs) comprise up to one fifth of skin tumors in dogs, rendering them the most common malignant skin neoplasm in this species [1, 2]. Cutaneous MCTs originate from the dermis and can extent into the underlying subcutis and muscles [3]. Literature since then adapted the term cMCTs. It took until 2007, when a separate subset of MCTs originating from subcutaneous tissues was first described [4]. Therapeutic decisions in canine cMCTs are based on the clinical condition of the dog, anatomic location of the tumor, staging, and histopathological differentiation, including grading, with the latter being one of the most crucial prognostic predictors [3–7]. A recent consensus proposal regarding diagnostic criteria and classification of MCTs has emphasized the importance of reporting the origin (cutaneous versus subcutaneous) and, for prognostication, to grade both tumor types [8]. The grading systems that are currently used to grade MCTs (3-tier Patnaik and 2-tier Kiupel) were both designed for grading cMCTs [3, 6]. However, since scMCTs were historically regarded as a subcutaneous variant of cMCTs, there are concerns whether some scMCTs were not inadvertently included when those grading systems were developed. In the absence of a grading system for scMCTs, negative prognostic factors that have been used to assess these tumors on histopathology are mitotic count (MC), multinucleation and infiltrative growth pattern [7, 9, 10]. The decision whether or not to grade scMCTs according to one of the existing grading systems was at the discretion of the pathologist. However, Sabattini and colleagues very recently studied the prognostic value of the Kiupel 2-tier grading in scMCT in dogs and concluded that it enables identification of aggressive biological behavior in scMCT cases, similar to cMCT cases [11]. Earlier, in terms of prognosis, it was believed that the majority of scMCTs exhibited a favorable prognosis compared to cMCTs, with extended survival times and lower metastatic rates and recurrence rates (4% and 8%, respectively) [7]. Later studies solely focusing on scMCTs, however, reported that a larger proportion of the scMCT cases might exhibit an aggressive biologic behavior [10, 12–14]. Our study aimed to describe signalment of dogs and histopathological features of scMCTs and cMCTs across a large dataset of canine MCTs of the skin. Our hypothesis centered on the comparability of histopathological features between scMCTs and cMCTs, anticipating similar characteristics in both groups.

## Methods

Anonymized pathology databases from 2 board-certified pathologists (A and B) from a single laboratory were screened and included reports of canine tissue samples from primary, secondary, and tertiary veterinary centers. Pathology reports that mentioned “skin” and “mast cells” between September 2020 and July 2023 were reviewed. Each report lacking the diagnosis of MCT or specific information on tumor origin (cutaneous versus subcutaneous) was excluded. Reports of dogs with more than one MCT, reports that mentioned incisional biopsy or reports that mentioned mast cells without histopathological diagnosis of MCT were excluded. Data retrieved from the database included information on signalment (breed, gender, and age), tumor dimension (in mm), and tumor origin (cutaneous or subcutaneous). For the purpose of the study, nine categories for MCT location were established: extremity, flank, perineal and genital region, back, head and neck, mammary gland, thorax, tail region, or buttock area). Histopathological features retrieved from the database were MC, bizarre nuclei and multinucleation, cytoplasmatic granules, eosinophil count, anisokaryosis, completeness of removal, histopathological grade (3-tier Patnaik and 2-tier Kiupel), and potential lymph node involvement. The MC was assessed in areas with the highest mitotic activity and reported as an absolute value, defined as the number of mitotic figures per 10 high-power fields (HPF) (x400, 2.37 mm²). For the purpose of the study, the presence of bizarre nuclei and multinucleation (in 10 HPF) was categorized in four categories (none or one bizarre nuclei/10 HPF, less than three bizarre nuclei/10 HPF, three or more bizarre nuclei/10 HPF, present but undefined). The presence of cytoplasmic granules had been categorized as a ‘small,’ ‘moderate,’ or ‘large’ number by each pathologist. Similarly, the number of eosinophils was categorized as ‘low’, ‘moderate’, or ‘high’. Pathologist A reported the presence and degree of anisokaryosis as a % of neoplastic cells of the total neoplastic population, that exhibits a two-fold variation in nuclear size. Pathologist B reported the presence and degree of anisokaryosis as none/mild/moderate or marked. Only data of pathologist A were used for statistical analysis regarding the presence of anisokaryosis to compare scMCTs versus cMCTs. The completeness of removal was described with the deep and horizontal margins taken into assessment. Margins were categorized as ‘incomplete’ if neoplastic cells extended to the surgeon-cut edge of the tissue in at least one plane of section. When information on lymph node metastasis was available, it was reported as either ‘present’ or ‘absent’ or classified as HN0 (non-metastatic), HN1 (pre-metastatic/suspected metastasis), HN2 (early metastasis), and HN3 (overt metastasis) when classification information was available [15].

### Statistical analysis

For categorical variables, subcutaneous MCTs and cMCTs were compared using the Cochrane Mantel Haenszel test with pathologists as stratification factor. The Breslow-Day Test for homogeneity of odds ratios was used to assess whether the comparison differed between the two pathologists. Analysis for numeric variables was based on the fixed effects model with pathologist as block factor. All analyses were performed at a significance level of 5%.

## Results

A total of 1685 histopathology records were reviewed, of which 1596 records in 1596 dogs contained information on tumor origin: 305/1596 (19.1%) scMCTs and 1291/1596 (80.9%) cMCTs. Pathologist A provided 1008 records of which 193/1008 (19.2%) scMCTs and 815/1008 (80.8%) cMCTs. Pathologist B provided a total of 588 records with 112/588 (19.1%) scMCTs and 476/588 (80.9%) cMCTs.

### Signalment

**Fig. 1:**
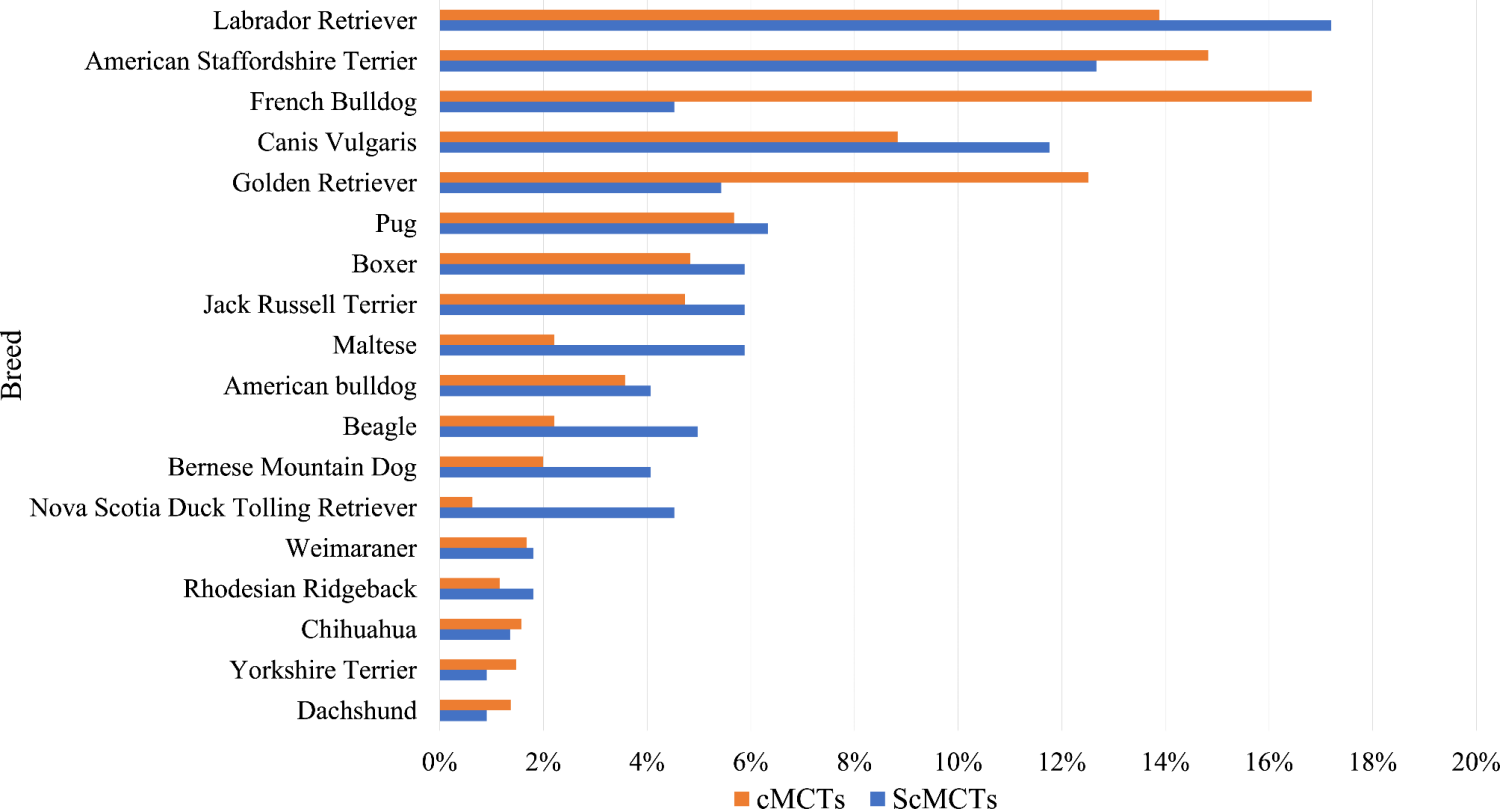
Most prevalent dog breeds (*n* = 18) with mast cell tumors (*n* = 1172 dogs). The 18 dog breeds in the study most prevalently diagnosed with a mast cell tumor, subdivided in subcutaneous mast cell tumors (scMCTs) (*n* = 221 dogs) versus cutaneous mast cell tumors (cMCTs) (*n* = 951 dogs)

Information regarding the age of dogs was available in 1533/1596 cases with a mean age of 7.63 ± 0.16 (SE) years for dogs diagnosed with scMCT and 7.57 ± 0.08 (SE) years for dogs diagnosed with cMCT (*P* = 0.7478). Gender was known for 1541/1596 dogs; 829/1541 (53.8%) were female and 712/1541 (46.2%) were male. Whereas female neutered dogs more often had scMCTs than cMCT (94/299, 31.4% versus 301/1241, 24.2%) male intact dogs more often had cMCTs (503/1541, 32.6% versus 84/299, 28.1%) but the difference was only borderline significant (*P* = 0.0495). A total of 105 different breeds were identified; breed was not mentioned in 103/1596 (6.5%) records. The 18 most common breeds made up 1172/1493 (78.5%) of all breeds and the breed distribution differed significantly between scMCTs and cMCTs (*P* < 0.0001) (Fig. 1).

### Tumor size, location, and completeness of removal

Information on tumor size of fixed tissue was available in 1071/1596 (67.2%) of cases: 220/305 (72.1%) scMCTs and 851/1291 (65.9%) cMCTs. On average, scMCTs were larger (17.87 cm³, [range 32–224.0 cm³]), compared to cMCTs (7.41 cm³, [range 8–266.32 cm³]) (*P* < 0.0001). Tumor location on the body was known for 1507/1596 (94.4%) MCTs: 290/305 (95.1%) scMCTs and 1217/1291 (94.3%) cMCTs and differed significantly (*P* = 0.0010) (Table 1). Subcutaneous MCTs were more often located in the thoracic region, extremities, flank, mammary gland and buttock while cMCTs were more often located in the head and neck, back, perineal and genital region, and tail. Information regarding completeness of excision was available in all cases, with scMCTs being more often (130/305; 42.6%) incompletely excised than cMCTs (233/1291; 18%) (*P* < 0.0001).

**Table 1:** Tumor location of mast cell tumors in dogs

Tumor location	scMCTs		cMCTs		Total (*P* = 0.0010)	
	*N*	%	*N*	%	*N*	%
	290	100	1217	100	1507	100
Head and neck	32	11.0	194	15.9	226	15.0
Thoracic region	32	11.0	120	9.9	152	10.1
Extremity	111	38.3	390	32.0	501	33.2
Flank	47	16.2	172	14.1	219	14.5
Back	7	2.4	35	2.9	42	2.8
Mammary gland	20	6.9	51	4.2	71	4.7
Buttock	29	10.0	113	9.3	142	9.4
Perineal and genital region	8	2.8	123	10.1	131	8.7
Tail	4	1.4	19	1.6	23	1.5

Tumor location of canine subcutaneous mast cell tumors (scMCTs), cutaneous mast cell tumors (cMCTs) and of the total group of mast cell tumors, displayed in absolute number (*N*) and relative percentage (%). A significant difference in tumor location on the body was observed between scMCTs and cMCTs (*P* = 0.0010)

### Histopathological features

Information on MC was available in all but 2 cases (303/305 scMCTs and 1291/1291 cMCTs) and was not different between scMCTs (1.63 ± 0.24 (SE)) and cMCTs (1.58 ± 0.12 (SE)) (*P* = 0.8490) (Table 2). Granule count did not differ between both tumor types (*P* = 0.0644). Subcutaneous MCTs more often had bizarre nuclei and multinucleation than cMCTs (36/283; 12.7% versus 12/1555; 7.7%) (*P* < 0.0001). Subcutaneous MCTs more often had a higher number of eosinophils compared to cMCTs (89/289; 30.8% versus 218/1269; 17.2%) whereas cMCTs more often had a moderate number of eosinophils compared to scMCTs (520/1269; 41.0% versus 61/289; 21.1%) (*P* < 0.0001). Subcutaneous MCTs more often had more than 10% anisokaryosis compared to cMCTs (25/188; 13.3% versus 76/811; 9.4% respectively) (*P* = 0.0269) (Table 3).

**Table 2:** Mitotic count of mast cell tumors in dogs

Mitotic count (*P* = 0.8490)	scMCTs		cMCTs		Total	
	*N*	%	*N*	%	*N*	%
	303	100	1291	100	1594	100
0	231	76.2	1031	79.9	1262	79.2
1	12	4.0	55	4.3	67	4.2
2	12	4.0	18	1.4	30	1.9
3	5	1.7	18	1.4	23	1.4
4	5	1.7	19	1.5	24	1.5
5	2	0.7	6	0.5	8	0.5
6	4	1.3	12	0.9	16	1.0
7	7	2.3	10	0.8	17	1.1
8	2	0.7	16	1.2	18	1.1
9	5	1.7	15	1.2	20	1.3
10	1	0.3	12	0.9	13	0.8
11	4	1.3	15	1.2	19	1.2
12	3	1.0	15	1.2	18	1.1
13	2	0.7	5	0.4	7	0.4
14	1	0.3	3	0.2	4	0.3
15	0	0.0	7	0.5	7	0.4
16	1	0.3	3	0.2	4	0.3
17	0	0.0	1	0.1	1	0.1
18	0	0.0	0	0.0	0	0.0
19	0	0.0	8	0.6	8	0.5
20	0	0.0	1	0.1	1	0.1
20+	6	2.0	21	1.6	27	1.7

Mitotic count of canine subcutaneous mast cell tumors (scMCTs), cutaneous mast cell tumors (cMCTs) and of the total group of mast cell tumors, displayed in absolute number (*N*) and relative percentage (%). No significant difference in mitotic count was observed between scMCTs and cMCTs (*P* = 0.8490)

**Table 3:** Histopathological features of mast cell tumors in dogs

Variables	ScMCTs		cMCTs		Total		*P*-value
	*N*	%	*N*	%	*N*	%	
**Bizarre nuclei and multinucleation**	283	100	1272	100	1555	100	< 0.0001
None or 1/10 HPF	247	87.3	1188	93.4	1435	92.3	
Yes, less than 3/10 HPF	0	0.0	10	0.8	10	0.6	
Yes, more than 3/10 HPF	1	0.4	60	4.7	61	3.9	
Yes, unspecified	35	12.4	14	1.1	49	3.2	
**Eosinophil number**	**289**	100	**1269**	100	**1558**	100	**< 0.0001**
High	89	30.8	218	17.2	307	19.7	
Moderate	61	21.1	520	41.0	581	37.3	
Low	139	48.1	531	41.8	670	43.0	
**Anisokaryosis** ^a^	**188**	100	**811**	100	**999**	100	= **0.0269**
Less than 5%	163	86.7	735	90.6	898	89.9	
Between 5–10%	0	0	0	0	0	0	
More than 10%	25	13.3	76	9.4	101	10.1	

Histopathological features of canine subcutaneous mast cell tumors (scMCTs), cutaneous mast cell tumors (cMCTs) and of the total group of mast cell tumors, displayed in absolute number (*N*) and relative percentage (%). Significant differences were observed between scMCTs and cMCTs

^*a*^*As % of neoplastic cells of the total neoplastic cell population*,* that exhibits a 2-fold variation in nuclear size (pathologist A)*

### Grading

The Kiupel grading system was applied to 292/305 (95.7%) scMCTs, and to all 1291/1291 (100%) cMCTs. The Patnaik grading system was only applied to 3/305 (0.9%) scMCTs and to all 1291/1291 (100%) cMCTs. Subcutaneous MCTs were more often assigned a Kiupel high grade than cMCTs (51/292; 17.5% versus 154/1291; 11.9%) (*P* = 0.0095). The odds of being assigned a Kiupel high grade in scMCTs was 1.578 higher than in cMCTs (95% confidence interval [1.116–2.232]) and was not different between both pathologists (*P* = 0.623). Of all tumors that were assigned a Kiupel grade, location on the body was known for 1494/1596 (93.6%) MCTs (277/305; 90.6% scMCTs and 1217/1291; 94.2% cMCTs). A significant difference in Kiupel grade between tumor locations was found in cMCTs (*P* = 0.016) and the total group of MCTs (*P* = 0.01) but not for scMCTs (*P* = 0.91). In both scMCTs and cMCTs, the perineal and genital region was the region that was most often assigned a Kiupel high grade (Table 4).

**Table 4:** Tumor location and Kiupel grading of mast cell tumors in dogs

	scMCTs				cMCTs				Total MCTs			
Tumor Location	Kiupel grade (*P* = 0.91)				Kiupel grade (*P* = 0.016)				Kiupel grade (*P* = 0.01)			
	Low		High		Low		High		Low		High	
	**N**	**%**	**N**	**%**	**N**	**%**	**N**	**%**	**N**	**%**	**N**	**%**
	228		49		1071		146		1299		195	
Head and neck	25	83.3	5	16.7	164	84.5	30	15.5	189	84.4	35	15.6
Thoracic region	25	83.3	5	16.7	106	88.3	14	11.7	131	87.3	19	12.7
Extremity	90	84.9	16	15.1	346	88.7	44	11.3	436	87.9	60	12.1
Flank	37	80.4	9	19.6	161	93.6	11	6.4	198	90.8	20	9.2
Back	6	85.7	1	14.3	35	100.0	0	0.0	41	97.6	1	2
Mammary gland	15	75.0	5	25.0	47	92.2	4	7.8	62	87.3	9	12.7
Buttock	21	80.8	5	19.2	100	88.5	13	11.5	121	87.1	18	12.9
Perineal and genital region	5	62.5	3	37.5	96	78.0	27	22.0	101	77.1	30	22.9
Tail	4	100	0	0.0	16	84.2	3	15.8	20	87.0	3	13.0

Association between tumor location on the body and Kiupel grading of canine subcutaneous mast cell tumors (scMCTs), cutaneous mast cell tumors (cMCTs) and of the total group of mast cell tumors (total MCTs), displayed in absolute number (*N*) and relative percentage (%). A significant difference in Kiupel grade between tumor locations was found in cMCTs (*P* = 0.016) and the total group of MCTs (*P* = 0.01), but not in scMCTs (*P* = 0.91)

### Lymph node metastasis

Information on lymph node status was available in 62/1596 (3.8%) reports. Of the lymph nodes that were submitted for histopathological evaluation, 23/62 (37.1%) were from scMCTs and 39/62 (62.9%) from cMCTs. Metastasis was absent in 7/23 (30.4%) of scMCTs and 20/39 (51.3%) of cMCTs (*P* = 0.1097) (Table 5).

**Table 5:** Lymph node classification of mast cell tumors in dogs

Metastasis (*P* = 0.1097)	scMCTs		cMCTs		Total	
	*N*	%	*N*	%	*N*	%
	23	100	39	100	62	100
HN1	2	8.7	1	2,6	3	4.8
HN2	1	4.3	1	2,6	2	3.2
HN3	3	13.0	4	10,3	7	11.3
Yes, but unspecified	10	43.5	13	33,3	23	37.1
Absent	7	30.4	20	51,3	27	43.5

Lymph node classification of canine subcutaneous mast cell tumors (scMCTs), cutaneous mast cell tumors (cMCTs) and of the total group of mast cell tumors, displayed in absolute number (*N*) and relative percentage (%), including classification according to Weishaar [15] when this was mentioned in the histopathology report. No significant differences were observed between scMCTs and cMCTs regarding metastasis (*P* = 0.1097)

## Discussion

Our study provides insights regarding the signalment and histopathological features of MCTs of the skin across a large canine population, and of all examined MCTs, nearly one-fifth was of subcutaneous origin. The results of our retrospective study confirm that, based on their histopathological features, the origin of the MCT, being subcutaneous or cutaneous, does matter. Differences between both tumor types were observed and when scMCTs were graded according to the Kiupel grading system, they were more often assigned a Kiupel high grade than cMCTs.

Boxer, French Bulldog, Weimaraner, Labrador Retriever, and Golden Retriever are well-known predisposed breeds for developing MCTs [16, 17]; these breeds were also among the 18 most prevalent breeds in our study. Breed distribution, however, was different for both tumor types when the 18 most prevalent breeds were compared. Subcutaneous MCTs were more often diagnosed in the Labrador Retriever, Maltese, Beagle, Bernese Mountain Dog, Boxer, and Nova Scotia Duck Tolling Retriever whereas the French Bulldog, Golden Retriever and American Staffordshire Terrier more often had cMCTs. Our study is the first to offer insights into the occurrence and breed distribution of scMCTs.

Cutaneous MCTs located in the genital area have been associated with a higher risk for Patnaik high-grade [18]. Other studies reported no worse prognosis for scMCTs or cMCTs located in the inguinal or perineal region [10, 19, 20]. The findings from our retrospective study indicate that anatomical location differed significantly between the two tumor types, with cMCTs more often located on the dorsal part of the body, and scMCTs more often on the ventral part. Kiupel grade was statistically different between tumor locations in cMCTs and the total group of MCTs. Mast cell tumors located in the perineal and genital region exhibited the highest frequency of Kiupel high grade. For scMCTs, the perineal and genital region was also the location that was most often assigned a Kiupel high grade. However, in scMCTs no significant difference in Kiupel grade between locations was found, which could be attributed to the lower sample size.

Whether these histopathology findings correlate with a worse outcome and prognosis remains to be elucidated by further prospective case-control studies.

In veterinary oncology, traditional histopathology still plays a major role. It does not only provide information about the type of tumor and the adequacy of excision, but it also assists in grading. Cell morphology, nuclear morphology, anisokaryosis, architecture, cellularity, stromal reaction, location, mitotic figures, edema, and necrosis are histopathological features evaluated in the Patnaik and/or Kiupel grading system [3, 6]. Mitotic count, bizarre nuclei and multinucleation, number of granules, number of eosinophils and anisokaryosis were histopathological features assessed in our retrospective study. Mitotic count and number of granules were not different between both tumor types whereas bizarre nuclei and multinucleation, number of eosinophils, and anisokaryosis were significantly different. The original Patnaik and Kiupel grading systems were specifically developed for cMCTs [3, 6] and both were not validated for grading scMCTs. In scMCT, the decision whether or not to apply the cMCT grading systems was left to the discretion of the pathologist; many of them would attribute a grade to scMCT and the majority would grade according to Kiupel. The two pathologists evaluating the scMCTs in this study graded all but 13 scMCTs according to Kiupel and only three according to Patnaik. If the Patnaik system is used to grade scMCTs, they would often be assigned grade II or higher because grade I tumors are confined to dermis and interfollicular spaces [3]. Unfortunately, the recent consensus that emphasizes the importance of reporting both Patnaik and Kiupel grade, was specifically proposed for cMCTs [21].

The original study validating the Kiupel 2-tier grading system in MCTs refers to an older study of the same research group that investigated the significance of tumor depth and tumor location for prognostic evaluation of cMCTs [6, 22]. In that study, however, scMCTs were considered as cMCTs isolated in the subcutis, thus being a subgroup of cMCT, and not as a distinct entity, as they are nowadays [8]. It is therefore not clear whether only true cMCTs were incorporated in the 95 cases on which the Kiupel grading system was established [6]. The question arises whether it would be interesting to reevaluate the 2-tier grading system in order to incorporate scMCTs, or to establish an independent grading system for both tumor types separately. After all, a recent consensus proposal emphasized the importance of grading both cMCTs and scMCTs for prognostication [8]. Another recent consensus regarding grading of MCTs solely focused on cMCTs [21]. Fortunately, a very recent study examined the prognostic utility of the Kiupel histologic grading system in 91 MCTs of the skin with six different growth model categories and explored the prognostic impact with emphasis on the growth model itself [11]. The authors demonstrated that the Kiupel grade had indeed a relevant prognostic value and that the Kiupel system could accurately identify any type of MCT with aggressive biologic behavior, including scMCTs [11]. In our study, both pathologists categorized a significantly higher percentage of scMCTs as Kiupel high grade compared to cMCTs (17.4% versus 11.9%). This grading system solely relies on cell morphology, in contrast to the Patnaik system, which also considers growth pattern and infiltration in surrounding tissues [3, 6]. Mast cell tumors are assigned a Kiupel high grade when any of the following criteria is present: ≥7 mitoses/10 HPFs, ≥ 3 multinucleated cells/10 HPFs, ≥ 3 bizarre nuclei/10 HPFs, and karyomegaly [6]. The results of our study show that around 10% of all MCTs exhibited a MC ≥ 7 (10.6% of scMCTs and 10.2% of cMCTs, respectively) with no difference between both types of tumors. Subcutaneous MCTs more often had anisokaryosis, and bizarre nuclei and multinucleation than cMCTs; unfortunately, the specific number /10 HPF was not always specified. However, the fact that anisokaryosis and bizarre nuclei and multinucleation were more often observed in scMCTs, may have contributed to the fact that scMCTs were more often assigned a Kiupel high grade in our study. Whether these histopathological features also correlated with a more aggressive behavior and worse clinical outcome could not be evaluated since information regarding clinical outcome was lacking.

It is well known that MCTs can metastasize to lymph nodes [23]. Unfortunately, in our study, only 3.9% of all MCTs had lymph nodes extirpated, and although there was no difference in metastatic rate between scMCTs and cMCTs, this number might be insufficient as a representative sample to draw definitive conclusions. Moreover, in the dataset it was not mentioned whether the excised lymph node was the locoregional (LRN) or sentinel lymph node (SLN). It emphasizes however the urgency for veterinarians to adopt a systematic approach to resecting LRN, and ideally, the SLN given the improved outcome when performing lymphadenectomy in dogs with biologically aggressive cMCTs [24].

Based on the histopathology reports, scMCTs were more often incompletely excised than cMCTs. This could be attributed to several factors. Firstly, the subcutaneous location could have made it harder to accurately determine surgical margins. Secondly, achieving deep margins during excision might have been more challenging with scMCTs. Moreover, surgeons might have based their surgical margins on previous literature with scMCTs exhibiting a more favorable prognosis. Finally, their bigger size might have raised concerns about subsequent closure, potentially prompting surgeons to opt for smaller margins during excision. The question arises whether incomplete excision had implications regarding the disease-free interval.

This study has several limitations. The retrospective nature of our study resulted in challenges related to standardization of histopathological data (bizarre nuclei and multinucleation, granule count, eosinophil count, anisokaryosis, metastasis and immunohistochemistry). Quantification using a range of values, and standardization would reduce observational bias in future studies. Functional outcome data on recurrence, disease-free interval, and survival time lacked, limiting the ability to establish direct correlations between histopathological findings and clinical prognosis. Obviously, such data is needed in a sufficient number of cases before the prognostic value of histopathological grading can be assessed.

## Conclusions

Subcutaneous MCTs represented a notable portion of the examined mast cell population, rendering them potentially more prevalent than previously recognized. They exhibited distinct histopathological characteristics including more anisokaryosis, bizarre nuclei, and multinucleation compared to cMCTs. In line, scMCTs were more frequently assigned a high grade than cMCTs when they were graded according to the Kiupel grading system. However, further studies are needed to determine if these histopathological differences correlate with clinical outcomes.

## Data Availability

The datasets used and/or analyzed during the current study are available from the corresponding author on reasonable request.

## Declarations

### Prior publication

Data have not been published previously.
